# A qualitative exploration of weight management during COVID‐19

**DOI:** 10.1111/cob.12512

**Published:** 2022-02-22

**Authors:** Meigan Thomson, Anne Martin, Emily Long, Jennifer Logue, Sharon A. Simpson

**Affiliations:** ^1^ MRC/CSO Social and Public Health Sciences Unit University of Glasgow Glasgow UK; ^2^ Lancaster Medical School University of Lancaster Lancaster UK

**Keywords:** barriers, behaviour change, COVID‐19, facilitators, weight loss, weight management

## Abstract

COVID‐19 has been associated with worse outcomes in people living with obesity and has altered how people can engage with weight management. However, the impact of risk perceptions and changes to daily life on weight loss has not been explored. This study aimed to examine how COVID‐19 and perception of risk interacted with weight loss attempts in adults participating in a behavioural weight management programme. Forty‐eight participants completed a semi‐structured interview exploring the impact of COVID‐19 on their weight management experience. Interviews were completed via telephone and analysed using a thematic approach. Reaction to perceived risk varied, but most participants reported the knowledge of increased risk promoted anxiety and avoidance behaviours. Despite this, many reported it as a motivating factor for weight loss. Restrictions both helped (e.g., reduced temptation) and hindered their weight loss (e.g., less support). However, there was consensus that the changes to everyday life meant participants had more time to engage with and take control of their weight loss. To the authors' knowledge, this is the first study to explore the impact of COVID‐19 on participation in a weight management programme started during the pandemic in the United Kingdom. Restrictions had varying impacts on participant's weight loss. How risk is perceived and reported to participants is an important factor influencing engagement with weight management. The framing of health information needs to be considered carefully to encourage engagement with weight management to mitigate risk. Additionally, the impact of restrictions and personal well‐being are key considerations for weight management programmes.

**What is already known about this subject**

- Obesity is linked to increased risk of worse COVID‐19 outcomes.
- COVID‐19 lockdowns and restrictions have caused changes in how the public interact with their environment, socially, and in their weight‐related behaviours

**What this study adds**

- Forty‐eight adults living with overweight, or obesity in the United Kingdom were interviewed about how COVID‐19 had impacted their weight loss journey while participating in an online behavioural weight loss programme
- The study found perception of risk, environmental and social changes, and personal well‐being all influenced participants' weight losses.

## 1 INTRODUCTION

Obesity has been identified as a leading risk factor for poorer COVID‐19 outcomes, with higher rates of hospitalization, complications, comorbidities, and mortality with increasing body mass index (BMI).^1^, ^2^ Those with obesity‐related comorbidities, such as elevated blood pressure and cholesterol, have had worse COVID‐19 outcomes when compared with BMI groups in the normal range.^3^, ^4^ Evidence from Public Health England has shown that 40% of adults gained weight during the COVID‐19 restrictions in the United Kingdom.^5^ Given the current increasing levels of obesity in the United Kingdom, with approximately 28% of adults living with obesity, and 36.2% overweight, this provides an added layer of complexity in tackling the public health pandemic of COVID‐19.^6^, ^7^

In response to the increased risk of worse COVID‐19 outcomes in those living with obesity, there have been calls for better policy and action to support people with obesity to lose excess body weight and thereby reduce their risk of poorer health outcomes.^4^, ^8^, ^9^ To support weight loss during a pandemic, it is key to consider how lifestyles have changed. Weight loss is commonly described as a journey, underlined by the Foresight report highlighting the myriad of influences on weight (i.e., policy and environmental, interpersonal, and intrapersonal factors).^10^ Within the United Kingdom, lockdown models (i.e., limitations on socializing and use of public services), food supply issues, and social distancing drastically changed how people lived.^11^, ^12^ Exploring how the COVID‐19 pandemic and the resultant changes to life, including how people socialized and interacted with their environment, affected weight loss journeys can provide novel insight into the interplay of health risk and perceptions, environment, and social changes.

Research has shown that COVID‐19 restrictions resulted in decreased levels of physical activity and increased emotional eating, sedentary behaviour, salt and sugar intake.^13^, ^14^, ^15^, ^16^ These behavioural changes were related to resource availability, emotional well‐being, and negative social changes (e.g., reduced social interactions, loneliness) associated with the imposed restrictions.^17^ For example, in the case of physical activity (PA), an observational study using remote monitoring through implanted cardiac devices found a significant decrease in PA during the first UK lockdown, with this gradually returning to baseline 3–4 weeks post‐lockdown.^13^

A study by Brown and colleagues, which surveyed 543 UK adults living with obesity in May and June 2020, found 55% reported having an unhealthier diet, 61% had reduced levels of PA, and many reported a decrease in general well‐being, with worse results in those who engaged in a weight management service before lockdown.^18^ A similar survey of 2002 UK adults also found a negative impact on eating and PA (in 56% of adults) and an increase in perceived barriers to weight management (i.e., reporting issues with motivation and control of diet).^19^ These changes were particularly evident in adults living with a higher BMI, which was associated with lower levels of PA and diet quality, and a higher frequency of overeating and snacking.

Although the above studies provide useful insights, in order to adequately support adults living with obesity or overweight to lose weight, it is important to understand their experiences during the pandemic. Qualitative studies can provide deeper insight into people's experiences, as well as the dynamics between factors influencing weight loss and well‐being. Grannell and colleagues interviewed participants of a weight management intervention in Ireland to assess the role of the pandemic on the sustainability of changes and the psychosocial impact of COVID‐19 on their weight loss. They found variety in responses to the pandemic in health behaviours, with low levels of awareness of the risk associated with obesity, and a negative impact on well‐being.^20^ This provides useful insights into how the pandemic influenced behaviour change from one hospital in Ireland. However, to our knowledge, there are no qualitative studies exploring the experience of those engaging in self‐referred online weight management programmes in the United Kingdom during COVID‐19. The aim of this study was to explore the impact of COVID‐19 and the associated restrictions on the experiences of participants in an online behavioural weight management programme. This provides unique insight into the impact of the pandemic on adults living with overweight and obesity who are actively engaging in weight loss attempts. Additionally, given increasing understanding that obesity and overweight are a risk factor for worse COVID‐19 outcomes, we wanted to understand whether this had an impact on people's motivation to lose weight or how they interacted with their physical and social environment, which could subsequently impact their weight loss success.

## 2 MATERIALS AND METHODS

### 2.1 Recruitment

Participants were recruited via the Second Nature online behavioural weight management programme. Participants were recruited from the paid arm in the United Kingdom. Second Nature is a 12‐week online programme that uses behaviour change techniques such as self‐monitoring, goal setting, and education on various lifestyle factors, including diet and PA. Participants were placed in an online group with other participants on the programme and a “health coach”. The “health coach” was a qualified dietician. The group allowed participants to share experiences with peers and sent queries to their health coach. This health coach led the group through the 12‐week programme by answering questions, facilitating discussion on barriers, facilitators, problem‐solving, and setting objectives.^21^ Throughout the programme, participants had access to their group anytime online. Participants were emailed information about the current study and a survey by the Second Nature programme staff once they signed up. Invitation emails were sent out in waves in October, November, and December 2020. Eligibility for the study consisted of being an adult (18+ years) who had recently enrolled in the Second Nature programme (0–2 weeks), with a BMI of 25 or above at the start of the programme. If participants were interested, they emailed the researchers for further information and/or to arrange participation in the study. Participants were compensated £20 for taking part in the interview. The data were collected as part of a wider mixed‐methods study investigating barriers and facilitators to weight loss, which consisted of data collected at three time points: an interview (of which the current study was a part of), social network data collection, and surveys (one at the beginning and one at the end of the project). Participants could opt to do either the surveys, the interview, or both. Social network data were collected at every time point.

### 2.2 Data collection

Participants completed a semi‐structured telephone interview with MT (PhD researcher) midway through participation in the programme (approximately 4–8 weeks). Data were collected as part of a wider interview study exploring barriers and facilitators to weight loss. Table 1 shows the COVID‐19‐specific section of the interview schedule. The interview schedule was developed following a review of the weight loss literature and discussions with people with lived experience. This approach allowed exploring the impact of COVID‐19 in different aspects of participants' lives (e.g., environment, social, personal well‐being, and risk perceptions) and how participants related this to their weight loss while in the programme.

**TABLE 1:** COVID‐19 questions from interview schedule

Question	
1.	How are you currently affected by the COVID‐19 situation regarding your weight loss?
2.	There have been some findings that people living with obesity have worse outcomes with COVID‐19 – Has this affected you in any way?
3	How much has your typical routine or activities changed because of the COVID‐19 restrictions?
4	Have the COVID‐19 measures changed who and/or how you socialize with people?
5	Is there anything else you would like to add about how COVID‐19 has impacted your weight loss journey so far?

All interviews were recorded using a portable digital recording device and took place at a mutually convenient time. Consent was obtained via email correspondence or recorded before commencement of the interview. Interviews lasted 60–90 minutes. Upon completion of the interviews, the audio files were uploaded, and interviews were transcribed verbatim.

### 2.3 Data analysis

Transcripts were analysed using thematic analysis.^22^ First, transcripts were read, and initial codes were generated according to the interview questions and research aims. This created a preliminary coding framework that was discussed with SS and AM. Transcripts were coded using Nvivo 12.^23^ Themes and sub‐themes were then generated both deductively and inductively and labelled using participant's words to keep themes true to the data. To reduce bias and increase dependability, 10% of the transcripts were reviewed by a second colleague (either SS or AM) and any nuances or discrepancies were discussed and resolved. Following the generation of themes, matrices were created to view responses and frequency of themes amongst the participants.

### 2.4 Reflexivity

To increase the rigour in the research, the lead author (MT) engaged in reflexive thinking throughout the research process and this should be considered while appraising the credibility of this study.^24^ The data for this study were collected as part of a larger mixed‐methods study investigating barriers and facilitators to weight loss in adults participating in behavioural weight loss programmes. The wider interview asked participants how various aspects of their lives related to their weight loss (i.e., interpersonal, intrapersonal, environmental, and programme factors). This may have influenced questions asked as well as MT's expectations within the interviews. However, the questions for the interviews were developed from a systematic search of the literature where factors influencing success were extracted and aligned with the social‐ecological model to identify what areas in a participant's life may impact their weight loss.^25^ Following the development of the interview schedule, two patient representatives were consulted on whether questions were clear, understandable, and holistic. During the interviews, MT also asked participants if they would like to add anything further about their experiences and if novel items emerged in the interviews this was explored. An effort was made to ensure spacing between interviews to allow for reflection and learning from each interview. To try and ensure participants felt comfortable, interviews were arranged at a mutually convenient time, building rapport, encouraging discussion through active listening, and the interview schedule was used flexibly to allow novel ideas to be explored. The study has followed the COREQ guidance for reporting qualitative research.^26^

## 3 RESULTS

### 3.1 Participant characteristics

Forty‐nine people participated in the interviews, with 48 included in the analysis, as one did not meet the eligibility criteria (BMI below 25 at the start of the programme). Most participants were female (83%; 40/48) with a mean age of 49.1 (SD: 10.2) (26–74) years and a mean BMI of 31.6 (SD: 4.8) (24.22–44.4) kg/m^2^ at the time of the interview. Participants were recruited across the four nations of the United Kingdom (England, Northern Ireland, Scotland, and Wales) where varying COVID‐19 rules and restrictions were in place. Participant information can be found in Table 2.

**TABLE 2:** Participant demographic information

Participant characteristic	*N* = 48
Age (mean, range, years)	49.09 (26–74 years)
Gender (% female)	83%
Ethnicity (% white)	95.8%
Employment Status (*n*, %)	26 (54.2%) – Employed full time 11 (22.9%) – Employed part‐time 3 (6.3%) – Unable to work due to illness or disability 3 (6.3%) – Retired 2 (4.2%) – Student 1 (2.1%) – Carer 1 (2.1%) – Supported through government‐funded pandemic job retention Scheme 1 (2.1%) No response given
Education (*n*, %)	3 (6.3%) – No further education from high school 15 (31.3%) – Qualification (not through college or university) 16 (33.3%) – Degree 13 (27.1%) – Higher degree 1 (2.1%) – Other
Household Income (*n*, %)	1 (2.1%) – £0–14 999 3 (6.3%) – £15 000–24 999 4 (8.3%) – £25 000–34 999 11 (22.9%) – £35 000–51 999 10 (20.8%) – £52 000–69 999 19 (39.6%) – £70 000 or above
Location in the United Kingdom (*n*, %) England Northern Ireland Scotland Wales	38 (79%) 1 (2%) 8 (17%) 1 (2%)
Interview BMI (mean, range)	31.6 (24.2–44.4 kg/m^2^)
Programme Status (at the end of the 12 weeks) (*N*, %)	17 (35.4%) completed the programme and are continuing to use it 10 (20.8%) completed and stopped using the programme 18 (37.5%) did not complete the programme 3 (6.3%) Lost contact
Weight Loss Category (at the end of the 12 weeks) (*N*, %)	7 (14.6%) – 10% or more weight loss 20 (41.7%) – 5% or more weight loss 14 (29.2%) less than 5% weight loss 2 (4.2%) – no weight change 5 (10.4%) – gained weight

Regarding their weight loss journey, participants had a mix of opinions and insights as to how the COVID‐19 pandemic and restrictions influenced their weight status, the decision to begin the programme, and progress during the programme. Table 3 provides an overview of the resultant themes from the analysis. Themes were grouped according to the relevant overarching themes of COVID‐19 risk, environment, interpersonal and personal well‐being.

**TABLE 3:** Overview of themes

Themes	Sub‐themes
COVID‐19 Risk	Recognizing weight as risk factor
	Hindering behaviours and emotions
	Motivating factor
Environment	Access to facilities
	Adapting activities
	Home
Interpersonal factors	Blocking support
	Social Responsibility
	Social Opportunities
Personal Well‐being	Uncertainty
	Time

### 3.2 COVID‐19 risk

#### 3.2.1 Recognizing weight as a risk factor

To understand the impact COVID‐19 had on weight loss, it was important to understand risk perception in this group. As noted in the introduction, excess body weight and comorbid conditions have been identified as a risk of worse COVID‐19 symptoms and outcomes. We, therefore, asked participants if this knowledge had impacted them in any way. Twenty‐six participants felt they were at increased risk due to their weight:

“I think that that…obviously, that is an issue, isn't it, and a concern. I think that that's my risk…that that would be my risk factors…if I was to contract COVID that [weight] would be my risk factor. That does…it does concern me.” (p154, female, 54 years)

While the remainder either viewed other health issues as higher risk factors were unsure, or felt there was little/no risk due to their weight.

“I think I'm more driven by the fact that age… but also my previous health assessment wasn't good” (p102, male, 54 years)

“…even though I'm overweight, I'd still see myself as low risk really” (p118, female, 34 years)

#### 3.2.2 Hindering behaviours and emotions

Most participants (44/48) reported the knowledge of increased risks negatively impacted either their behaviours and/or emotions.

Emotional impacts included feelings of shame about their weight and fear/anxiety about catching COVID‐19:

“if I did catch COVID, you know, like, being overweight was the factor that made me, like, you know, die from it, it feels a bit embarrassing… you would feel, yeah, a bit ashamed that that was…because you're fat, so that was the thing that tipped you over the edge.” (p114, female, 42 years)

Emotions related to risk also seemed connected to how the health messages were shared with the participant, with more fear‐inducing information causing greater negative responses:

“It was like a bomb underneath me… I got a letter which basically said, see nobody, speak to nobody, go to your room, hide. Don't breathe the same air as everybody else, and I thought, oh my god, it was absolutely terrifying” (p118, female, 34 years)

For many, this fear and anxiety response impacted participants' weight loss. Some reported avoiding social and environmental interactions, which limited how much they could engage with behaviours associated with weight loss (e.g., PA, shopping for healthy foods), which, in turn, they reported as hindering their ability to fully focus on their weight loss:

“I've shied myself away because I feel like I'm more at risk, I haven't done the exercise that I should have done, that's weighing on my mind quite a bit” (p110, female, 37 years)

#### 3.2.3 Motivating factor

However, half of the participants (24/48) reported COVID‐19 risk was a motivating factor for their weight loss journey and health:

“It definitely added to my worries but also to my motivation of losing weight… I want to lose weight because having COVID with my weight is dangerous” (p119, female, 34)

### 3.3 Environment

#### 3.3.1 Access to facilities

Weight‐related behaviours (i.e., diet and PA) were impacted by the restrictions through changing access to different environments. Reduced access to facilities acted as both a barrier and facilitator to participants. Difficulty in accessing healthy food for meals (i.e., due to shortages of food in supermarkets) and exercise facilities (e.g., gym, swimming pool) acted as barriers:

“I remember going to the supermarket, couldn't find anything really to eat… it was just a case of buying anything that would make some sort of a meal. And then the personal training stopped as well and the swimming.” (p125, female, 38)

While others found the closure of certain facilities reduced temptation and made it easier to lose weight:

“I actually found that easier for me because I wasn't tempted by you know, restaurants and things like that. And I did actually lose weight over the lockdown rather than gaining it, so that was actually quite good.” (p109, female, 35)

#### 3.3.2 Adapting activities

Participants explained how they had to adapt their usual activities to the COVID‐19 restrictions. Some tried to navigate restrictions to maintain a sense of normality by maintaining habits that likely hindered progress:

“We can still go down the pub, interestingly, actually, so the pubs have to serve food. And we do very much use the pub in the village an awful lot. And last week, we went twice and ate both times because that's the only way that you can go for a drink now.” (p137, female, 37)

While others discussed socializing and how they engaged with their environment had ultimately changed, all participants reported an increased engagement with green space generally and specifically for social activities:

“I think before coronavirus, we'd sort of meet people outdoors, but we'd also do a lot of meeting in restaurants and that side of thing. I think the balance is now more meeting up with people outdoors” (p124, male, 40)

### 3.4 Home

There was less consensus on the impact of spending more time at home on weight loss. Some participants found this worked as a barrier to their progress by reducing their PA, taking care of family members (e.g., children, elderly), adapting to more time at home, and having greater access to temptations:

“I think what happens is as well, when you're working from home, you work that little bit longer, and you sit still for longer. You've got people, maybe, that are at home, bringing you a drink, whereas you'd have maybe gone for a walk round at work, so I've probably not been as, I've just been more sedentary” (p120, female, 50)

While others felt being at home more gave them greater control over what and when they ate, which supported their diet:

“You can choose what's in your environment, no one's going to turn up with a birthday cake or a biscuit or invite you out for a pint” (p138, male, 45)

### 3.5 Interpersonal

#### 3.5.1 Blocking support

Participants also spoke about how their relationships had been affected by the COVID‐19 restrictions and how this impacted their weight loss. Reduced support was identified as a barrier, both for emotional and instrumental support, creating difficulties in focusing on the programme and their goals:

“I haven't really had anybody to offload the problems that I've been facing… it has impacted probably my mental health as well, my focus on being able to give 100 per cent to my diet” (p122, female, 37)

“I used to go and have a wee kickabout a couple of nights a week, which I'm not able to do now, at all… so yeah, I think COVID has limited my ability to do stuff with other folk” (p103, male, 60)

#### 3.5.2 Social responsibility

Beyond COVID‐19 restrictions inhibiting social support, many felt their social responsibilities changed. Some spent more time caring for children, parents, and other family members, while others spoke about having a responsibility to protect those they deemed as more vulnerable creating emotional stress in their lives:

“Whenever I saw my mother, it would be a hug and kiss every time I leave, and I've not given her a hug or a kiss since March, so I find it difficult and stressful, and my mother will take the opinion, oh, I would rather see you than not see you, and I'm just like, yeah, but I'd rather not kill you than kill you.” (p131, male, 50)

Often this facilitated adherence to COVID‐19 restrictions (i.e., social distancing) and minimizing contact with those they perceived as vulnerable. Participants reported reducing contact with those they were close with to be an emotional strain, which sometimes compromised their focus on weight loss. Many also reported their responsibility to protect those who were vulnerable in their lives motivated them to focus on improving their health and immunity and supported engagement with healthful behaviours.

#### 3.5.3 Social opportunities

Participants also discussed how fewer social opportunities facilitated their weight loss. Without the pressure of adhering to social norms, participants felt it was easier to follow their diet proposed by the Second Nature programme and they had fewer temptations to navigate:

“… the social norm to have a coffee with a cake every day when you go out is difficult. And the fact that I'm in COVID and I'm not going out to have coffee and cake makes it so much easier” (p104, female, 57)

### 3.6 Personal well‐being

#### 3.6.1 Uncertainty

Participants reported that the uncertainty around COVID‐19 and associated restrictions had substantial impacts on participants' well‐being. The uncertainty of how long restrictions and COVID‐19 would last caused disruption to many participants experiences and resulted in emotional eating or low motivation:

“it's mental health and what's going on in your life at the moment and, you know, the uncertainty of, like, COVID and, you know, we've just been told that we're going into tier three. And it, that's really depressing and it's, I almost, like, reach for something to put in my mouth” (p110, female, 37)

#### 3.6.2 Time

Even with the uncertainty, changes, and emotional turmoil brought by COVID‐19, all participants reported that they felt they had more time to engage with the programme and take control of their weight loss:

“I think COVID has probably helped, the fact that I'm stuck in and I've got the time to do it [Second Nature programme]…I don't have all the meetings and things that I used to attend, for example. I think if I was still juggling, it would have been harder to meal prep, it would have been harder to cook.” (p104, female, 57)

“I've gone from that sense of everything being out of my control to literally the opposite, I've taken control. I've prioritised different things for me, I'm exercising every single morning… And then during the day I'm getting up, I'm giving myself permission to walk around the house, to talk about something, do a job that's completely unrelated and then come back to my office and continue. Those sorts of things have radically changed my outlook and now I feel much more in control.” (p132, female, 44)

## 4 DISCUSSION

Overall, we found heterogeneity in the impact COVID‐19 had on participants' weight loss efforts. The social, work, and environmental limitations imposed by COVID‐19 restrictions could also create a differing experience in the weight loss programme outside of the programme's or participant's control. Reaction to such restrictions during participation could also be variable depending on the individual's motivations, mood, changes to their circumstances, and readiness/ability to make changes. However, this illustrates the variety of influencing factors we need to consider when supporting participants to change their behaviour, especially in circumstances where they have limited control over their environment.

Participants in this research reported variable experiences of how the COVID‐19 pandemic and restrictions in the United Kingdom affected their weight loss journey. Over half of the participants felt they were at increased risk to COVID‐19 due to their weight, with the remainder either viewing other health issues as higher risk, being unsure about their risk levels, or feeling there was little/no risk due to their weight. Despite the differing views on perceived risk, most participants still reported negative behaviours and emotions (e.g., fear, avoidance), and felt that the increased risks of COVID‐19 associated with excess body weight acted as a motivational factor for weight loss. COVID‐19 restrictions both helped and hindered participants' weight loss. Weight loss journeys were supported through reduced opportunities to engage in tempting facilities (e.g., restaurants and cafes), having more control over their environments, encouraging more use of and socialization in the outdoors, and giving participants more time to focus on their diet and weight loss programme. While barriers incurred by COVID‐19 included the uncertainty surrounding the longevity of restrictions, lack of access to food and exercise facilities, being at home more, blocked social support, and increased feelings of social responsibility.

Future health campaigns would benefit from understanding the varying responses to health messaging, particularly how to promote positive motivation. Despite many participants reporting COVID‐19 risk as a motivating factor, 44/48 participants still reported emotional and behavioural drawbacks of the news, such as fear, anxiety, shame, and avoidance behaviours. These hindering reactions could have wider ramifications for supporting psychological well‐being and coping throughout their weight loss. It is, therefore, crucial to explore how to avoid such negative reactions while promoting positive ones (i.e., encourage positive engagement with weight management to mitigate risk associated with obesity), and to understand why some who experienced the hindering emotions and behaviours became motivated while others did not. One theory that may offer insights into the disparity of the health messaging becoming a motivating factor in the current study is the protection motivation theory. This theory suggests that a combination of our threat appraisal (i.e., COVID‐19 risk and weight status, and how these link) alongside our coping appraisal (i.e., self‐efficacy and skill level to mediate the threat) influence our behaviours.^27^, ^28^ With the participants in this study, a discrepancy in threat appraisal or coping appraisal could have led to differing reactions. Both could have influenced how health messages were delivered to participants.^29^ In the field of weight management, while health risks of excess weight are common knowledge, it is often the early diagnosis that prompts behavioural and lifestyle changes, suggesting the risk of ill‐health is not perceived as a strong enough threat to warrant change.^30^, ^31^ Furthermore, Youngs et al. found patients given a pre‐diabetes diagnosis were not motivated to change until they formally had a type 2 diabetes diagnosis and attributed this to lack of understanding of the label and self‐efficacy/support on how to implement changes.^32^ Other research has shown the importance of how health messages are phrased and delivered to encourage behavioural change.^29^ For example, one study found personal and contextual factors influenced how participants living with obesity interpreted health messaging and ultimately reacted.^33^ The authors reported for adults living with obesity, phrasing health messages to convey the risks and complexity of obesity while minimizing stigma were desired by participants.

This study also showed how environmental restrictions impacted participants' weight loss journeys. Limited access to facilities like cafes, restaurants, and others acted to support and make the weight loss journey easier, while the unavailability of healthy food in shops and lack of access to fitness facilities hindered weight loss. However, participants often adapted their activities to include more use of green space, thereby promoting PA. The shift to working and being at home more often meant some participants felt a barrier to their progress due to reduced movement throughout the day, managing other family members, and having greater access to temptations while others felt they gained more control over their diet and PA. This echoes the results of Avery and colleagues (2021) study who found access to sugary foods and feeling bored inhibited weight loss in participants in a weight management programme while others reported having more free time enabled better meal planning and time to cook.^15^

Social relationships and formats for socializing were changed greatly by the COVID‐19 restrictions, which participants reported as an emotional toll impacting their weight loss. Participants reported having less support to offload problems and to facilitate change. Research has shown social support is key to successful weight loss.^34^, ^35^ For example, a study by Kumanyika and colleagues highlighted how vital support during weight loss can be: they found participants who took part in a programme paired with a family or friend had greater success in their weight loss journeys.^36^

The uncertainty related to COVID‐19 restrictions took a toll on participants' well‐being. Participants reported feeling anxious due to the changes, suggesting that a clear set of expectations, potential outcomes, and guidance would reduce COVID‐19 anxiety and associated impact on weight loss. However, participants also reported having more time, due to less social interactions and working from home, improved their well‐being and confidence in the weight loss journey. Having time to take control of their diet and PA increased feelings of self‐efficacy. Participants reported this as the largest facilitator and previously the largest barrier to weight loss, suggesting weight management services could benefit participants by including innovative ways to make weight loss programmes fit better into people's routines. This could be achieved by managing participants' expectations of how long, and the level of commitment required each week, to attain their weight goals. While the extent to which participants require this type of guidance may differ based on lifestyles, knowledge, and self‐efficacy, studies have shown clear guidance and meal planning are linked to success, which arguably may be due to saving time in knowledge acquisition and weight loss tasks.^37^, ^38^

### 4.1 Study limitations

While this study presents novel insight into the COVID‐19 pandemic and its effect on weight management, there are several limitations. Firstly, COVID‐19 restrictions were variable and changed throughout the interview period. Participants experienced different restrictions depending on where they resided, and often the rules had just changed or were about to change. The study, therefore, reflects a changing environment rather than a steady impact of COVID‐19 restrictions. The participants in this study had all paid to take part in the weight loss programme and chose to contact the research team to take part, which may limit the range of views gathered as part of this research. Similarly, the sample overall had a BMI in the lower obesity range, and there was a lack of ethnic diversity and men in the study. In terms of methodology, the use of telephone interviews may have impacted the data we collected, as rapport with the interviewer is more difficult to achieve than in a face‐to‐face setting. However, the interviewer MT did not identify any difficulties gaining rapport with participants. This approach may also have supported participants to be more candid due to feelings of anonymity. Research has shown that when telephone and in‐person interviews are compared, they are of similar duration and detail, and participants report finding it easier to focus on the voice rather than the face of the researcher and feeling less judged and uninhibited when on the telephone.^39^, ^40^, ^41^

### 4.2 Study strengths

The study provides novel insights into how the COVID‐19 pandemic affected weight loss journeys of participants in a behavioural weight loss programme in the United Kingdom. This is useful for making suggestions on how to manage public health in any future pandemics as well as for general weight management services. Such suggestions can be used for general weight management practice and policy and can arguably also be applied to groups who face similar limitations outside of COVID‐19. For example, those who are trying to lose weight or services who are trying to engage with harder‐to‐reach groups (i.e., those with reduced access to resources and socializing, with little time, and those with ill‐health).

### 4.3 Implications for policy and practice

Based on the findings of this research, considerations for policy and weight management services are summarized in Table 4. These are implications for policy and practice, which are applicable to weight loss during COVID‐19 but are useful for consideration in a post‐COVID era. Our key suggestions from this work include framing messages in a clear and accessible way to support understanding of risk, supporting skill development and perception of control over how behaviours and interaction with the environment can impact weight/health behaviours, and supporting understanding of how behaviours can impact those around them. These suggestions could apply in a post‐COVID setting to those with reduced social contact (e.g., due to location), understanding health risks associated with weight and behaviour, and in future pandemics.

**TABLE 4:** Suggestions for policy and practice based on participant experiences

Participant Experiences	Implication
Differing levels of recognition of weight status as a risk factor	Clearer information is needed regarding how weight is related to health and how weight loss can be beneficial
Negative behavioural and emotional reaction to risk messages	Health messages need to be framed in a constructive and accessible way following best practice.^42^, ^43^ Providing information on how to be safe and how to reduce risk. Supporting participants by providing guidance, skills, and tools to motivate change^44^
Motivated by knowledge of risk	
Facility Access	Providing guidance on how to use the local environment to support weight loss.
Level of control over the environment	Developing strategies with participants to increase perception of control over the environment and how to navigate social situations
Need for support	Fostering emotional and instrumental support for those taking part in weight loss programmes from those in their social network.
Protecting other's health	Educating people on how their lifestyle decisions can impact others health.
Negative consequences of uncertainty	Providing clear and explicit information and guidance on what is currently happening could happen and is happening next. Building resilience and developing coping strategies for uncertain times and situations.
Time	Encouraging participants to consider how much time they have for making changes. Making goals, skill development, and changes achievable in the time and with the resources they have. Delivering information and suggesting ways to reduce time demand in participants lives (e.g., meal planning, shopping lists)

## 5 CONCLUSIONS

The current study found that knowledge of obesity as a risk factor for worse COVID‐19 outcomes had varying effects on participants' weight loss. The framing of health information regarding increased risk to COVID‐19 needs to be considered carefully during the pandemic and post‐COVID‐19 times. The study showed participants had varying perceptions of risk, which could be attributed to how the health information was shared and their understanding of the relationship between obesity and health. There was also differing positive (i.e., increased motivation) and negative (i.e., feelings of shame, avoidance behaviours) to risk. Further research exploring these differing reactions would be useful in understanding how to promote health behaviour change in post‐COVID times. Similarly, participants' experiences were variable with the consensus that the emotional demands of the pandemic due to uncertainty and social changes acted as an obstacle. Largely, participants felt that changes in access to facilities and having more time to understand and engage with their weight management supported weight loss.

## CONFLICT OF INTEREST

JL received personal fees from Novo Nordisk. No other competing interests.

## STATEMENT OF ETHICS

Ethical approval was granted by the College of Social Sciences ethics committee at the University of Glasgow (Application number: 400190202). All participants provided informed written or verbal consent to undertake this research. Consent for publication of data was provided by participants as per good research practice.

## AUTHOR CONTRIBUTIONS

All authors contributed to the study design and development of the interview questions. Jennifer Logue facilitated contact with Second Nature. Meigan Thomson conducted interviews, analysis, and drafted the manuscript. Anne Martin and Sharon A. Simpson reviewed the analysis and results were discussed with Meigan Thomson. All authors reviewed and contributed to the manuscript.

## Data Availability

The data generated and analysed during the current study are not at present publicly available as they have been collected as part of a PhD project. However, following publication of the PhD thesis and an embargo period they will be made freely available from a data archive. An update will be posted on the University of Glasgow's enlighten research data repository under the lead author's profile (www.researchdata.gla.ac.uk).
